# Complete Genome Sequence of *Clostridioides difficile* Epidemic Strain DH/NAP11/106/ST-42, Isolated from Stool from a Pediatric Patient with Diarrhea

**DOI:** 10.1128/genomeA.00923-17

**Published:** 2017-09-21

**Authors:** Egon A. Ozer, Alan R. Hauser, Dale N. Gerding, Robyn O. Espinosa, David W. Hecht, Larry K. Kociolek

**Affiliations:** a Northwestern University Feinberg School of Medicine, Chicago, Illinois, USA; b Loyola University Chicago Stritch School of Medicine, Maywood, Illinois, USA; c Edward Hines, Jr. VA Hospital, Hines, Illinois, USA; d Ann & Robert H. Lurie Children’s Hospital of Chicago, Chicago, Illinois, USA

## Abstract

We report here the complete genome sequence of *Clostridioides difficile* strain DH/NAP11/106/ST-42, which is now the most common strain causing *C. difficile* infection among U.S. adults. This strain was isolated from the stool from a hospitalized pediatric patient with frequent relapses of *C. difficile* infection.

## GENOME ANNOUNCEMENT

Recent *Clostridium difficile* (newly reclassified as *Clostridioides difficile* [1, 2]) surveillance data presented by the U.S. Centers for Disease Control and Prevention suggest that epidemic strain ribotype 106 (via PCR ribotyping) is now the predominant strain causing *C. difficile* infection (CDI) among U.S. adults (3, 4). This ribotype has also been identified as group DH (by restriction endonuclease analysis [REA]) and NAP11 (by pulsed-field gel electrophoresis) (5). We previously reported the predominance of REA group DH in our single-center pediatric cohort (6). We report here the complete genome sequence of *C. difficile* strain DH/NAP11/106/ST-42 isolated from the stool from a hospitalized pediatric patient.

In 2012, CDI was diagnosed by *tcdB* PCR in a hospitalized teenage patient with diarrhea. Saved stool was subsequently cultured anaerobically, and the isolate was identified by REA as a previously uncharacterized subtype within group DH, similar to DH7. This patient developed eight subsequent CDIs over a 3-year period. Stool samples from three of these subsequent CDIs were available for culture, and all were identified as the same DH subtype, suggesting frequent relapse with this particular strain.

The MICs (in micrograms per milliliter) as measured via the agar dilution method (7, 8) were 0.25 for metronidazole, 0.5 for vancomycin, 0.0039 for rifaximin, 0.125 for fidaxomicin, 0.25 for surotomycin, 4 for clindamycin, and 2 for moxifloxacin. Thus, this isolate was susceptible to metronidazole (8), vancomycin (9), rifaximin (10), and moxifloxacin (8) and intermediately susceptible to clindamycin (8). Susceptibility breakpoints are not yet established for surotomycin and fidaxomicin.

Genomic DNA was extracted and pooled from 28 subcultures of this isolate using the BiOstic bacteremia DNA isolation kit (Mo Bio Laboratories, Inc., Carlsbad, CA). Libraries for PacBio sequencing on the RSII (Pacific Biosciences, Menlo Park, CA) were prepared by shearing DNA with g-TUBES (Covaris), targeting an average fragment size of 10 kb. The SMRTbell template preparation kit (Pacific Biosciences) was used to ligate hairpin adapters required for sequencing to the fragmented DNA. Libraries were size selected using the BluePippin (Sage Science, Beverly, MA) and sequenced using PacBio’s P6-C4 chemistry and 240-min movies. PacBio raw data were corrected and assembled using HGAP assembler (SMRT Analysis 2.3.0), Canu assembler version 1.2, and Celera assembler version 8.2. The assemblers were run at default. Assemblies were assessed for inconsistencies and misassembly using Nucmer whole-genome alignments and Circleator plots (GC skew). The genome was assembled into a single contig with average read coverage of 205-fold. The GC content was 28.62%. The contig was circularized using Circlator version 1.5.1. No plasmids were identified. The final assembly was polished using Quiver (SMRT Analysis 2.3.0). Indel errors were corrected using Pilon version 1.21 using 301-bp paired-end reads generated on an Illumina MiSeq system, with an average read coverage of 120-fold. The genome length after circularization and indel correction was 4,087,127 bp. On-instrument adapter trimming was performed. Annotation was performed by the NCBI Prokaryotic Genome Annotation Pipeline. The sequence contained 3,562 coding sequences. The isolate sequence type (ST) was identified as ST-42 by multilocus sequence typing (see http://pubmlst.org/cdifficile).

### Accession number(s).

This whole-genome shotgun project has been deposited in DDBJ/ENA/GenBank under the accession number CP022524. The version described in this paper is the first version.
